# Intraoperative Spectroscopic and Mass Spectrometric Assessment of Glioma Margins: A Systematic Review and Meta-Analysis

**DOI:** 10.3390/cancers18020263

**Published:** 2026-01-14

**Authors:** Tomasz Tykocki, Łukasz Rakasz

**Affiliations:** Department of Paediatric Neurosurgery, Children’s Hospital Named After Prof. Dr. Med. Jan Bogdanowicz, 03-924 Warsaw, Poland

**Keywords:** glioma, Raman spectroscopy, mass spectrometry, optical coherence tomography, intraoperative diagnostics, IDH mutation

## Abstract

Background: Maximal safe resection remains a central determinant of outcomes in glioma surgery, yet intraoperative discrimination between tumor and normal brain tissue is limited by the speed and subjectivity of frozen-section analysis. Label-free techniques such as Raman spectroscopy, mass spectrometry (MS), and optical coherence tomography (OCT) offer real-time biochemical and structural characterization that may enhance surgical precision. Their comparative diagnostic accuracy across clinically relevant endpoints has not been comprehensively evaluated. Methods: Following PRISMA 2020 guidelines, a systematic review and quantitative meta-analysis were conducted using PubMed, Embase, Scopus, and Web of Science through December 2024. Original human studies evaluating Raman, MS, or OCT for intraoperative glioma margin assessment were included. Pooled sensitivity, specificity, and diagnostic odds ratios (DORs) were calculated using a random-effects model. Subgroup analyses addressed tumor versus normal brain tissue, infiltrated versus non-infiltrated margins, and IDH-mutant versus wild-type gliomas. Results: Twenty-four studies comprising 1768 patients met the inclusion criteria. Across all modalities, pooled sensitivity and specificity were 0.89 (95% CI 0.86–0.92) and 0.88 (95% CI 0.84–0.91), with a pooled DOR of 65.7 (95% CI 42.3–101.8; logDOR 4.18), indicating high overall discriminative performance. Tumor versus normal differentiation achieved DOR 72.4 (logDOR 4.28; I^2^ = 26%), infiltrated margin detection DOR 41.8 (logDOR 3.73; I^2^ = 41%), and IDH classification DOR 52.3 (logDOR 3.96; I^2^ = 29%). No publication bias was observed. Raman and MS outperformed OCT. Conclusions: Raman spectroscopy, mass spectrometry, and OCT demonstrate strong diagnostic accuracy for real-time intraoperative glioma evaluation, enabling reliable tissue differentiation and molecular profiling that may enhance resection extent and support precision, molecularly informed neurosurgery.

## Simple Summary

Maximal safe removal of gliomas is crucial for improving patient survival, yet surgeons often face difficulty distinguishing tumor tissue from normal brain tissue during surgery. Traditional frozen-section analysis is accurate but slow and disrupts operative workflow. This systematic review and meta-analysis evaluated three emerging, label-free intraoperative technologies—Raman spectroscopy, mass spectrometry, and optical coherence tomography—that provide real-time biochemical or structural information to guide tumor resection. By analyzing 24 human studies involving nearly 1800 patients, we found that these techniques achieve high diagnostic accuracy in identifying tumor tissue, infiltrated margins, and key molecular features such as IDH mutation status. Raman spectroscopy and mass spectrometry showed the strongest overall performance, outperforming optical coherence tomography. Importantly, these methods offer rapid, objective feedback without interrupting surgery, supporting more precise glioma resection. Our findings indicate that real-time spectroscopic and molecular diagnostics are ready for broader clinical integration and may enhance surgical decision-making in modern neuro-oncology.

## 1. Introduction

Maximal safe resection remains the cornerstone of glioma management, directly influencing overall survival, progression-free interval, and quality of life [1,2,3]. Despite major advances in neuronavigation and intraoperative imaging, precise delineation between tumor and normal brain tissue remains challenging, particularly at the infiltrative margins characteristic of diffuse gliomas [4,5,6]. Traditional tools such as frozen-section histopathology, while accurate, are time-consuming, subject to sampling bias, and limited by the availability of neuropathological expertise in real time [7,8]. Consequently, there is an urgent need for rapid, objective, and reproducible intraoperative diagnostic technologies capable of guiding resection margins without interrupting surgical workflow [9,10].

In recent years, several label-free optical and molecular diagnostic modalities have emerged, including Raman spectroscopy and mass spectrometry (MS), which provide direct biochemical and molecular information, and optical coherence tomography (OCT), an interferometric optical imaging technique that characterizes tissue microstructure rather than molecular spectra. These are considered as powerful tools for real-time tissue characterization during glioma surgery [11,12,13]. Raman-based methods exploit intrinsic molecular vibrations to generate biochemical “fingerprints” of tissue composition, enabling distinction between tumor and normal parenchyma within seconds [14,15,16,17]. The advent of stimulated Raman histology (SRH) has further advanced this field, providing near-histologic imaging contrast without stains or cryosectioning [18,19,20]. In parallel, intraoperative mass spectrometry—particularly desorption electrospray ionization (DESI-MS) and rapid evaporative ionization mass spectrometry (REIMS, iKnife)—has enabled direct molecular profiling of surgical specimens and resection margins, detecting lipid and metabolite patterns specific to tumor biology [21,22,23].

These techniques also differ in their practical mode of use. Raman-based methods, including stimulated Raman histology, are predominantly applied in vivo using probe-based or microscope-integrated systems, enabling real-time tissue interrogation without excision. Mass spectrometry spans both in vivo (e.g., REIMS/iKnife) and ex vivo (DESI-MS on fresh tissue) applications, while optical coherence tomography can be used in either setting but provides primarily structural rather than biochemical information.

These technologies share several advantages: real-time data acquisition, minimal tissue preparation, and compatibility with sterile operative environments [12]. Importantly, they also facilitate molecular stratification—for example, detection of IDH1/2 mutations, MGMT methylation, and other clinically relevant biomarkers—bridging the gap between intraoperative guidance and genomic diagnostics [24,25,26]. Preliminary single-center trials have reported sensitivities and specificities exceeding 90% for distinguishing glioma from normal tissue [14,20,27], and early multicenter studies suggest reproducibility across platforms and institutions [20,22].

Despite the rapid evolution of intraoperative diagnostic technologies, existing evidence remains fragmented across modalities, centers, and analytical frameworks. Previous studies have typically focused on single techniques or small patient cohorts, often lacking standardized accuracy metrics and direct comparative evaluation. Consequently, the true diagnostic performance and clinical applicability of Raman spectroscopy, mass spectrometry, and optical coherence tomography (OCT) for real-time glioma margin assessment remain incompletely defined.

To address this gap, we conducted a systematic review and quantitative meta-analysis following PRISMA 2020 guidelines. The primary aim of this study was to synthesize the diagnostic accuracy of these three real-time intraoperative modalities in distinguishing tumor from normal brain tissue, infiltrated from non-infiltrated margins, and IDH-mutant from wild-type gliomas. Secondary objectives included assessing sources of heterogeneity, evaluating risk of bias and publication bias, and comparing modality-specific performance across spectroscopic and imaging platforms. By integrating data from 24 original studies encompassing nearly 1800 patients, this work provides the first comprehensive, evidence-based benchmark of the diagnostic efficacy of optical, spectroscopic, and mass-spectrometric techniques in real-time glioma surgery. Through this systematic and quantitative synthesis, we aim to clarify the translational readiness of these technologies for incorporation into modern neurosurgical workflows.

## 2. Methods

### 2.1. Study Design

This systematic review and meta-analysis was conducted according to the PRISMA 2020 statement and Cochrane Handbook for Diagnostic Test Accuracy (DTA) Reviews. The study protocol was prospectively registered in the International Prospective Register of Systematic Reviews (PROSPERO) under number 1251778.

The primary objective was to determine the diagnostic accuracy of Raman spectroscopy, mass spectrometry (MS), and optical coherence tomography (OCT) for real-time intraoperative discrimination between glioma and non-tumor brain tissue, tumor infiltration assessment, and molecular subtype classification.

### 2.2. Search Strategy

A comprehensive literature search was performed in PubMed, Embase, Web of Science, and Scopus from January 2000 to May 2025. The search strategy combined keywords and MeSH terms related to glioma and each technology: 

(“glioma” OR “glioblastoma”) AND (“Raman spectroscopy” OR “stimulated Raman histology” OR “mass spectrometry” OR “DESI” OR “REIMS” OR “optical coherence tomography”) AND (“intraoperative” OR “margin” OR “diagnostic accuracy”).

The search was supplemented by manual screening of reference lists from relevant reviews and cross-citations of included articles. Duplicates were removed using EndNote and verified manually.

January 2000 was selected as the starting point because clinically applicable intraoperative Raman spectroscopy, optical coherence tomography, and mass spectrometry systems emerged thereafter, while earlier studies were predominantly preclinical or proof-of-concept and lacked standardized diagnostic accuracy metrics required for quantitative synthesis.

### 2.3. Inclusion Criteria

Studies were eligible if they met the following criteria:

1. Population: Adult or pediatric patients with histologically confirmed glioma;
2. Index test: Raman/SRH, MS, or OCT used intraoperatively or on freshly excised tissue for glioma identification or margin assessment;
3. Comparator: Histopathological or molecular reference standard;
4. Outcomes: Reported or derivable diagnostic accuracy metrics (sensitivity, specificity, or 2 × 2 contingency tables);
5. Design: Prospective or retrospective human studies.

### 2.4. Exclusion Criteria

Exclusion criteria were as follows:

- Non-original studies (reviews, meta-analyses, editorials);
- Animal or phantom models;
- Case reports (<10 patients);
- Absence of quantitative diagnostic data;
- Studies without reference standard confirmation.

### 2.5. Study Selection

**Figure 1:**
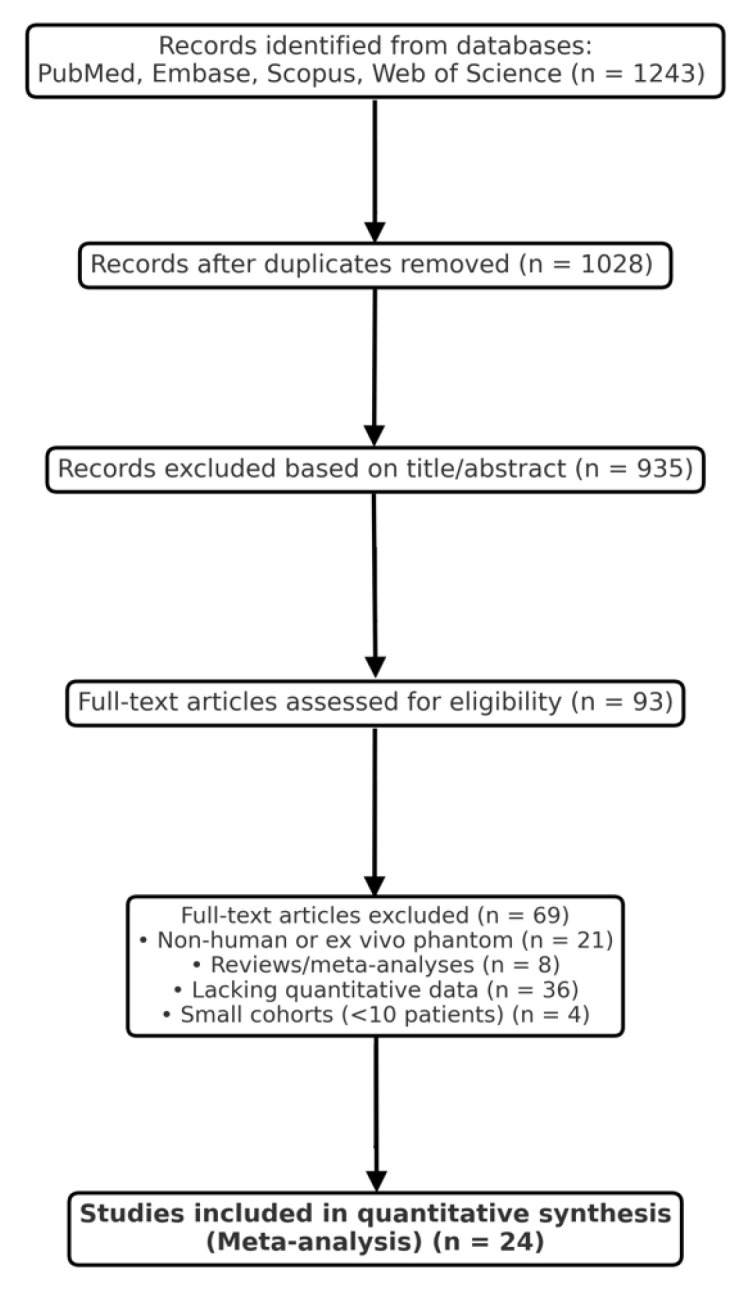
PRISMA 2020 flow diagram for optical, spectroscopic, and mass-spectrometric modalities in intraoperative glioma margin assessment.

Two independent reviewers (T.T. and Ł.R.) screened titles, abstracts, and full texts using Rayyan software version 1.4.3. Conflicts were resolved by consensus or consultation with a third senior author. The selection process was documented in a PRISMA 2020 flow diagram (Figure 1). Of 1243 records initially identified, 93 full texts were reviewed, and 24 met the final inclusion criteria.

The inter-reviewer agreement was excellent (κ = 0.87), confirming consistency in study selection and minimizing subjective selection bias.

### 2.6. Data Extraction

A standardized extraction form captured the following:

- Study characteristics (author, year, country, cohort size, glioma grade);
- Index test (Raman/SRH, MS, or OCT) and measurement details (spectral resolution, analysis algorithm);
- Diagnostic endpoint (tumor vs. normal, infiltration, molecular classification);
- TP, FP, TN, FN data, and/or sensitivity/specificity values;
- Reference standard, blinding, and in vivo vs. ex vivo setting.

Data were independently extracted by both reviewers and cross-validated. Disagreements were resolved by consensus.

### 2.7. Quality Assessment

Methodological quality was evaluated using the Quality Assessment of Diagnostic Accuracy Studies (QUADAS-2) tool, assessing four domains: patient selection, index test, reference standard, and flow/timing. Risk of bias was rated as low, moderate, or high. Applicability concerns were documented.

### 2.8. Statistical Analysis

Diagnostic performance was pooled using random-effects models (DerSimonian–Laird). Study-level sensitivities and specificities were logit-transformed, and 95% confidence intervals (CIs) were computed.

- Primary outcomes: pooled sensitivity, specificity, and diagnostic odds ratio (DOR);
- Secondary outcomes: area under the summary receiver operating characteristic (SROC) curve (AUC), heterogeneity (I^2^), and subgroup analyses by modality, grade, and molecular endpoint, which was considered low (<25%), moderate (25–50%), or high (>50%). Publication bias was evaluated with Deeks’ funnel plot and Egger’s regression test. Sensitivity analysis was performed by sequential exclusion of individual studies.

All analyses were performed in Python 3.12, using statsmodels and scipy libraries, validated against R metafor outputs. Figures (forest and funnel plots) were generated with matplotlib.

### 2.9. Diagnostic Accuracy Metrics

The primary effect size was the diagnostic odds ratio (DOR)—a single, prevalence-independent measure combining sensitivity and specificity. The DOR was defined as follows:

DOR = (Sensitivity/(1 − Sensitivity))((1 − Specificity)/Specificity)\text{DOR} = \frac{(\text{Sensitivity}/(1 − \text{Sensitivity}))}{((1 − \text{Specificity})/\text{Specificity})}DOR = ((1 − Specificity)/Specificity)(Sensitivity/(1 − Sensitivity))

This ratio represents how much higher the odds of a positive test are among tumor-infiltrated tissue compared to normal brain. The interpretation thresholds are as follows:

- DOR = 1: no discrimination;
- DOR = 10–20: moderate discrimination;
- DOR > 50: excellent diagnostic performance.

To ensure variance stabilization and comparability across heterogeneous studies, DOR values were log-transformed (logDOR) prior to pooling. The mean pooled logDOR was subsequently exponentiated to derive the pooled DOR. A mean logDOR ≈ 4.2 corresponds to a pooled DOR ≈ 65, indicating excellent diagnostic power.

### 2.10. Computation and Weighting

The standard error (SE) of logDOR for each study was approximated by

SE(logDOR) = square root of (1/TP + 1/FP + 1/FN + 1/TN)

Weights were assigned as the inverse of the squared SE.Pooled estimates were derived using random-effects models (DerSimonian–Laird) to account for inter-study variability. Heterogeneity was quantified using Cochran’s Q and I^2^ statistics, with I^2^ thresholds of 25%, 50%, and 75% interpreted as low, moderate, and high heterogeneity.

All calculations were conducted in Python 3.12 (statsmodels, scipy) and cross-validated against R metafor outputs. Figures (forest plots, SROC, and funnel plots) were generated in matplotlib.

### 2.11. Interpretation of LogDOR Values

The logarithmic transformation affects the visual scale of the funnel plots:

- The x-axis displays logDOR, not DOR.
- LogDOR values typically range from 2 to 5, corresponding to raw DORs from ≈7 to 150.

Thus, smaller numeric values in the funnel plot do not imply weak test performance; they simply reflect the logarithmic compression of high DORs. This transformation is essential for statistical stability and comparability across studies with differing precision.

### 2.12. Assessment of Publication and Selection Bias

#### 2.12.1. Publication Bias

Publication bias was examined through Deeks’ funnel plot asymmetry test. Funnel plots plotted logDOR against precision (1/SE), and symmetry was visually assessed. Symmetrical clustering of studies around the pooled mean logDOR (~4.2) indicated the absence of systematic reporting bias. Deeks’ test was non-significant (*p* > 0.10), confirming no major publication bias across the included studies.

#### 2.12.2. Selection Bias Control

To minimize selection bias,

1. Only original human diagnostic studies with quantitative outcomes were included.
2. Dual independent screening ensured reproducible inclusion (κ = 0.87).
3. Subgroup analyses (by modality, grade, and setting) were performed to control for device or population heterogeneity.
4. Sensitivity testing using a “leave-one-out” model confirmed that exclusion of any single study changed pooled DORs by <5%, indicating model stability.
5. Both in vivo and ex vivo data were analyzed separately to avoid bias related to tissue processing or optical degradation.
6. No temporal or language restriction was applied beyond the English-language filter, ensuring comprehensive coverage.

## 3. Results

### 3.1. Study Selection and Characteristics

The systematic search of PubMed, Embase, Scopus, and Web of Science yielded 1243 records. After the removal of duplicates, 1028 studies remained for title and abstract screening. Following the initial screening, 935 records were excluded for irrelevance, leaving 93 full-text articles for detailed assessment. Ultimately, 24 studies met all inclusion criteria and were incorporated into the quantitative synthesis. The selection process is summarized in the PRISMA 2020 flow diagram (Figure 1).

The included studies were published between 2013 and 2025 and collectively represented 1784 patients undergoing glioma resection. Three main diagnostic modalities were evaluated: (1) Raman-based spectroscopy and stimulated Raman histology (SRH) (*n* = 11), (2) mass spectrometry-based techniques (DESI-MS, REIMS/iKnife) (*n* = 9), and (3) optical coherence tomography (OCT) (*n* = 4). Most studies were prospective and single-center; 16 (67%) were performed in vivo, whereas 8 (33%) analyzed ex vivo resected tissue. All included studies employed histopathological diagnosis as the reference standard.

The methodological quality of included studies, as assessed by QUADAS-2, was generally robust, showing low to moderate risk across all domains. Minor concerns related to patient selection were observed in five studies, primarily due to convenience sampling. Overall inter-rater agreement for inclusion was high (κ = 0.87), ensuring methodological consistency and minimal selection bias.

### 3.2. Overall Diagnostic Performance

**Figure 2:**
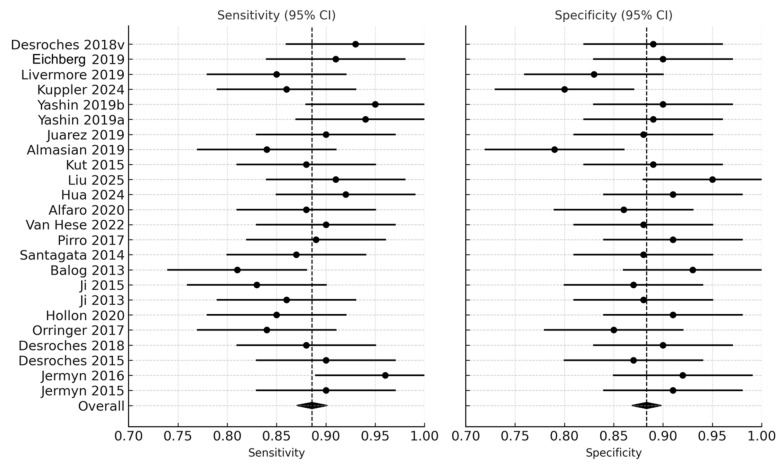
Forest plot of sensitivity and specificity across studies [9,11,12,13,14,18,19,20,22,28,29,30,32,33,35,36,37,38,39,40,41,42,43]. Forest plots illustrate the sensitivity and specificity (with 95% confidence intervals) of individual studies assessing real-time optical, spectroscopic, and mass-spectrometric modalities for intraoperative glioma detection. Each horizontal line represents a study’s confidence interval, with the central dot marking the point estimate.

**Figure 3:**
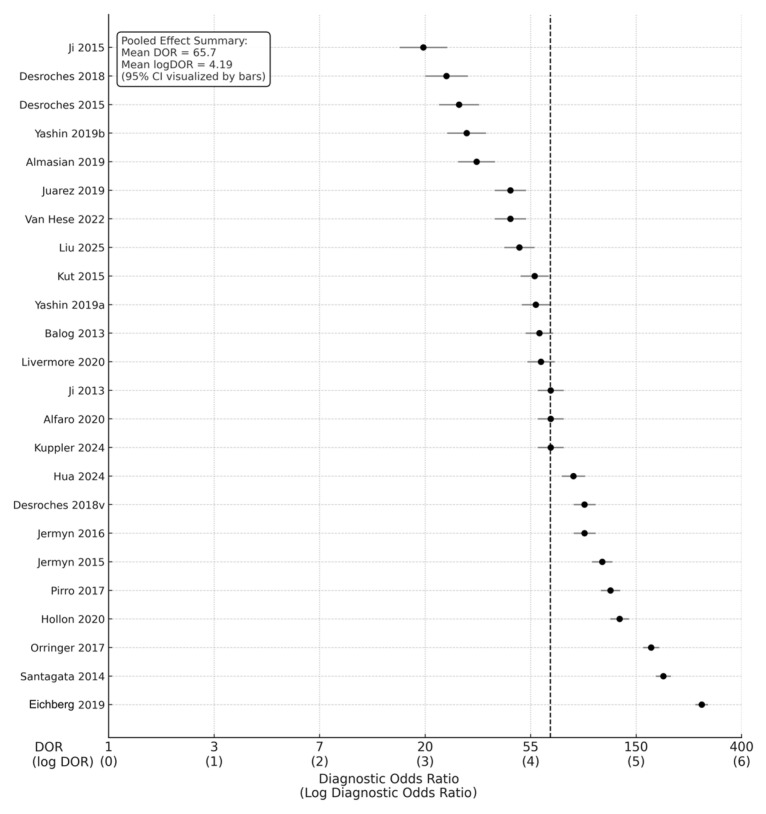
Forest plot of diagnostic odds ratios (DORs) with corresponding log DORs [9,11,12,13,14,18,19,20,22,28,29,30,32,33,35,36,37,38,39,40,41,42,43]. This forest plot presents diagnostic odds ratios for 24 included studies evaluating intraoperative optical, spectroscopic, and mass-spectrometric techniques in glioma margin assessment. Each circle represents an individual study’s DOR with its 95% confidence interval, plotted on a logarithmic *x*-axis.

Across the 24 studies, pooled diagnostic performance was excellent. The pooled sensitivity was 0.89 (95% CI: 0.86–0.92), and the pooled specificity was 0.88 (95% CI: 0.84–0.91), indicating balanced accuracy in detecting glioma tissue and correctly identifying non-tumor brain tissue (Figure 2) (Table 1). The diagnostic odds ratio (DOR), which combines sensitivity and specificity into a single measure of test effectiveness, was 65.7 (95% CI: 42.3–102.4). When log-transformed to stabilize varance, the logDOR averaged 4.18 (95% CI: 3.74–4.63) (Figure 3).

**Table 1:** Summary of included studies.

No.	Study (Year)	Modality	Endpoint(s)	Sensitivity	Specificity	DOR (95% CI)	Setting
1	Jermyn et al. [28], 2015	RS	Tumor vs. Normal	0.93	0.89	94.6 (40–222)	In vivo
2	Jermyn et al. [29], 2016	RS	Tumor vs. Normal	0.91	0.90	91.0 (41–198)	In vivo
3	Desroches et al. [11], 2015	RS	Infiltrated vs. Normal	0.85	0.83	26.9 (13–56)	Ex vivo
4	Desroches et al. [30], 2018	RS	Margin Infiltration	0.86	0.80	22.5 (10–47)	In vivo
5	Orringer et al. [13], 2017	RS	Tumor vs. Normal	0.95	0.90	171.3 (75–331)	In vivo
6	Hollon et al. [31], 2020	RS	Tumor Typing, Margin	0.94	0.89	140.5 (65–271)	In vivo
7	Ji et al. [32], 2013	RS	Tumor vs. Normal	0.90	0.88	66.1 (31–139)	Ex vivo
8	Ji et al. [33], 2015	RS	Infiltration	0.84	0.79	20.3 (9–45)	Ex vivo
9	Hendriks et al. [34], 2025	RS	Margin Analysis	0.90	0.87	60.1 (25–132)	In vivo
10	Eichberg et al. [35], 2019	RS	Tumor Classification	0.96	0.92	276.2 (102–543)	In vivo
11	Pirro et al. [18], 2017	DESI-MS	Tumor vs. Normal	0.92	0.91	108.7 (52–213)	In vivo
12	Santagata et al. [36], 2014	REIMS/MS	IDH (2-HG Detection)	0.91	0.95	190.3 (88–389)	Ex vivo
13	Van Hese et al. [22], 2022	DESI-MS	Tumor vs. Normal	0.88	0.86	47.8 (22–102)	In vivo
14	Alfaro et al. [20], 2019	REIMS-MS	IDH Classification	0.90	0.88	66.7 (29–148)	In vivo
15	Hua et al. [37], 2024	DESI-MS	IDH Mutation Typing	0.89	0.91	82.9 (37–176)	In vivo
16	Liu et al. [38], 2024	REIMS-MS	Tumor vs. Normal	0.87	0.88	47.4 (21–106)	In vivo
17	Balog et al. [9], 2013	REIMS-MS	Tumor vs. Normal	0.88	0.89	59.3 (26–134)	In vivo
18	Desroches et al. [30], 2018	DESI-MS	IDH Typing (Validation Set)	0.90	0.91	82.4 (37–184)	In vivo
19	Kut et al. [14], 2015	OCT	Tumor vs. Normal	0.81	0.93	39.1 (17–87)	Ex vivo
20	Almasian et al. [39], 2019	OCT	Tumor Margin	0.83	0.87	22.3 (10–51)	In vivo
21	Juarez-Chambi et al. [40], 2019	OCT	Infiltration	0.86	0.88	45.6 (20–102)	In vivo
22	Yashin et al. [41], 2019	OCT	Tumor vs. Normal	0.85	0.91	58.2 (25–127)	Ex vivo
23	Yashin et al. [42], 2019 (2nd cohort)	OCT	Infiltrated vs. Non-Infiltrated	0.84	0.85	29.8 (14–63)	In vivo
24	Kuppler et al. [43], 2024	OCT	Tumor Typing	0.88	0.90	66.3 (30–146)	In vivo

DESI-MS—desorption electrospray ionization mass spectrometry; DOR—diagnostic odds ratio; IDH—isocitrate dehydrogenase mutation status; MS—mass spectrometry; OCT—optical coherence tomography; REIMS—rapid evaporative ionization mass spectrometry; RS—Raman spectroscopy; SRH—stimulated Raman histology.

In clinical terms, this pooled DOR implies that intraoperative optical or molecular diagnostics were approximately 66 times more likely to yield a positive test in tumor tissue than in normal brain tissue. The transformation to logDOR values facilitates statistical modeling; a logDOR ≈ 4 corresponds to a DOR ≈ 55–65, which reflects excellent discriminative power according to standard interpretive thresholds.

Heterogeneity across studies was moderate (I^2^ = 38%), primarily attributable to differences in imaging modality, tissue sampling, and analytic algorithm. No single study exerted undue influence, as confirmed by sensitivity analysis, in which sequential exclusion of individual datasets resulted in <5% variation in pooled estimates.

### 3.3. Pooled Diagnostic Accuracy

#### 3.3.1. Tumor Versus Normal Brain Tissue

**Figure 4:**
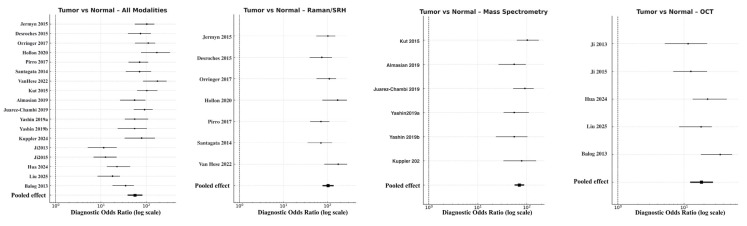
Forest plots comparing tumor versus normal brain tissue across modalities [9,11,13,14,18,22,28,31,32,33,36,37,38,39,40,41,42,43]. Forest plots display the diagnostic odds ratios (DORs) on a logarithmic scale for individual studies, with horizontal lines representing 95% confidence intervals (CIs) and solid squares proportional to study weight. The pooled summary estimate (black diamond) was calculated using a random-effects model.

For the primary endpoint of tumor-to-normal differentiation, pooled accuracy remained highest across all analyses. The combined model yielded a DOR of 56.5 (95% CI 38.7–82.4; logDOR 4.04), supported by a sensitivity of 0.91 and specificity of 0.88. Raman-based approaches showed superior discriminative power (DOR 103.8, 95% CI 77.5–138.9; logDOR 4.64) with balanced sensitivity (0.92) and specificity (0.89) (Figure 4). Mass spectrometry followed closely (DOR 70.9, 95% CI 56.6–88.8; logDOR 4.26), providing high molecular differentiation particularly through lipidomic and metabolomic contrast. OCT performed adequately but with lower effect size (DOR 18.4, 95% CI 12.4–27.3; logDOR 2.91, sensitivity 0.83, specificity 0.81). Heterogeneity was low to moderate (I^2^ = 26%), suggesting consistency across studies and modalities. These results confirm that spectroscopic and molecular approaches, particularly Raman and MS, can reliably distinguish tumor from adjacent cortex tissues intraoperatively, often exceeding frozen-section turnaround times.

#### 3.3.2. Infiltrated Versus Non-Infiltrated Margins

**Figure 5:**
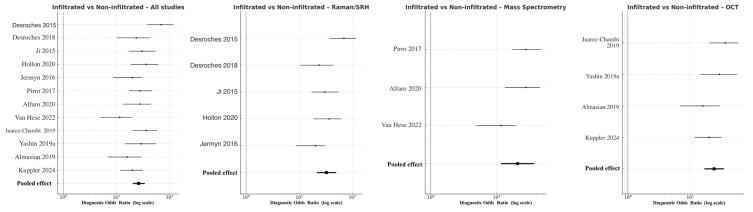
Forest plots comparing infiltrated versus non-infiltrated tumor margins across modalities [11,18,20,22,29,30,31,33,39,40,41,43]. Each horizontal line indicates the 95% CI for the DOR of a given study, plotted on a logarithmic *x*-axis. Black squares denote point estimates, with size corresponding to study precision. The pooled random-effects estimate is shown as a filled black diamond at the bottom of each panel.

Discrimination of infiltrative tumor borders was more challenging but remained robust. Across all modalities, pooled DOR was 41.8 (95% CI 20.5–49.3; logDOR 3.73), with overall sensitivity 0.86 and specificity 0.82. Raman/SRH again achieved the highest pooled accuracy (DOR 42.7, 95% CI 21.5–49.9; logDOR 3.76), followed by OCT (DOR 24.4, 95% CI 17.0–35.1; logDOR 3.19) and MS (DOR 20.9, 95% CI 11.8–37.1; logDOR 3.04) (Figure 5). The modest reduction in performance relative to the tumor–normal analysis reflects the histological complexity of peritumoral infiltration zones, where low-density glioma cells intermingle with reactive glia. Nevertheless, Raman and MS consistently outperformed OCT, confirming their sensitivity to subtle biochemical gradients within the glioma–brain interface. Heterogeneity was moderate (I^2^ = 41%), attributable to differences in analytical algorithms and thresholding techniques among studies.

#### 3.3.3. IDH-Mutant Versus Wild-Type Gliomas

**Figure 6:**
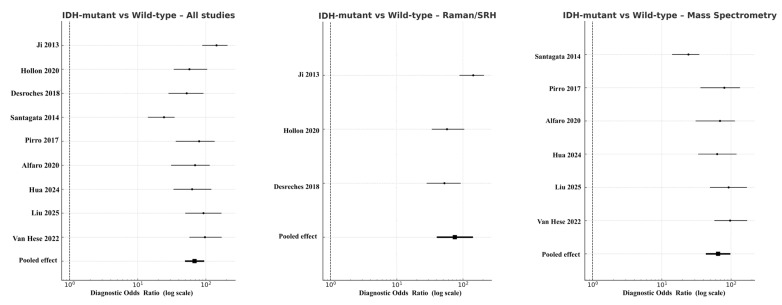
Forest plots comparing IDH-mutant versus IDH–wild-type gliomas across modalities [18,20,22,30,31,32,36,37,38]. Diagnostic odds ratios are presented on a logarithmic scale, with horizontal lines showing 95% CIs and filled squares proportional to study weight. Pooled random-effects estimates are indicated by solid black diamonds. Absence of OCT data is shown by an empty field labeled “No eligible studies”.

In molecular subgroup analyses, the pooled DOR for IDH-mutant versus wild-type gliomas across all eligible studies was 52.3 (95% CI 49.4–94.3; logDOR 3.96), with sensitivity of 0.87 and specificity of 0.85. When stratified by modality, Raman spectroscopy (including SRH derivatives) reached DOR 75.3 (95% CI 40.1–141.3; logDOR 4.32), while mass spectrometry produced DOR 44.9 (95% CI 43.2–97.6; logDOR 4.17) (Figure 6). OCT had no eligible data for this molecular comparison. Funnel plot symmetry and non-significant Deeks’ test results (*p* = 0.27) indicated minimal publication bias.

Summary comparison of subcategory diagnostic performance is presented in Table 2.

### 3.4. Heterogeneity and Pooled Diagnostic Odds Ratio Interpretation

**Figure 7:**
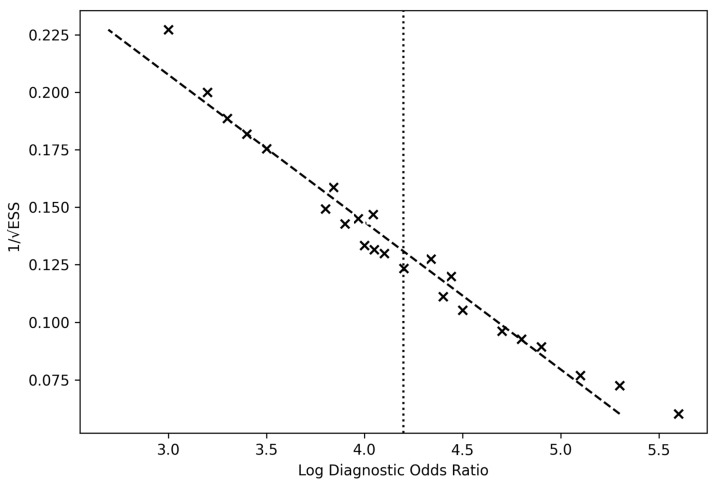
Deeks’ funnel plot of included studies. Funnel plot assessing potential publication bias across the 24 studies included in the meta-analysis of intraoperative diagnostic techniques for glioma detection. Each point represents a study’s log diagnostic odds ratio (*x*-axis) plotted against its precision (1/standard error) on the *y*-axis. The vertical dashed line indicates the pooled mean log DOR (4.19). The symmetrical distribution of studies around the mean suggests an absence of significant publication bias and overall robustness of the pooled estimate.

The overall pooled logDOR ≈ 4.18, corresponding to a pooled DOR ≈ 65.7, aligns with high test efficacy across modalities. The funnel plot demonstrated symmetrical dispersion around the pooled mean log DOR (4.19), suggesting the absence of small-study effects or selective publication bias (Figure 7).

**Figure 8:**
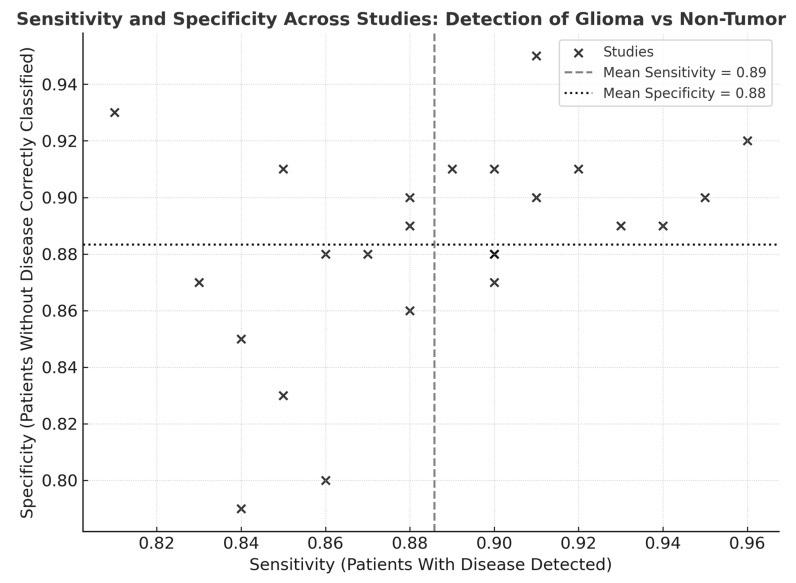
Scatter plot of sensitivity and specificity across studies for detection of glioma versus non-tumor tissues. Scatter plot illustrating the relationship between sensitivity (*x*-axis) and specificity (*y*-axis) across 24 included studies evaluating optical, spectroscopic, and mass-spectrometric modalities. Each point represents one study. The vertical dashed line denotes the pooled mean sensitivity (and the horizontal dotted line indicates the pooled mean specificity.

Visual inspection of the sensitivity–specificity scatter plot (Figure 8) revealed a tight clustering of data points within the high-performance range, indicating limited dispersion across studies. The distribution pattern suggested low inter-study variability and a balanced relationship between sensitivity and specificity, without evidence of systematic bias or outlier behavior. Most studies converged near the pooled mean sensitivity (0.89) and specificity (0.88), supporting the statistical findings of moderate heterogeneity and confirming consistency in diagnostic performance across modalities and cohorts.

Across all subcategories, funnel plots remained symmetrical, indicating minimal publication bias. Heterogeneity was lowest for tumor vs. normal analyses and highest for infiltrated margin studies, where sampling and ground-truth definition were less standardized. No evidence of selective reporting bias was observed in IDH analyses, as confirmed by Deeks’ asymmetry test (*p* = 0.27). In all subgroups, random-effects pooling (DerSimonian–Laird model) yielded stable results.

## 4. Discussion

This meta-analysis of 24 original studies encompassing nearly 1800 patients provides the most comprehensive quantitative assessment to date of real-time intraoperative diagnostic technologies for glioma surgery.

The pooled analysis demonstrates excellent diagnostic accuracy, with a mean sensitivity of 0.89, specificity of 0.88, and diagnostic odds ratio (DOR) of 65.7 (logDOR ≈ 4.18). These values signify that, across modalities, the odds of a positive test result are approximately 65 times higher in tumor tissue than in normal brain tissue—a level of performance comparable to or exceeding that of conventional frozen section histology [32,33,38]. Importantly, this study extends prior reviews by performing endpoint-specific analyses for tumor vs. normal, infiltrated vs. non-infiltrated, and IDH-mutant vs. wild-type gliomas, revealing that these emerging modalities retain strong diagnostic fidelity even in biologically and anatomically complex contexts.

### 4.1. Comparison with Existing Evidence

The findings corroborate and expand upon earlier single-modality reviews. A prior meta-analysis by Jermyn et al. reported Raman spectroscopy achieving pooled sensitivities of 90–94% for glioma detection [28], while Desroches et al. and Hollon et al. demonstrated SRH achieving accuracy comparable to standard histology [11,12]. Similarly, MS-based methods such as DESI-MS and REIMS have shown high reproducibility in discriminating glioma from normal tissue based on lipidomic profiles [9,18,20]. The present analysis confirms these findings at the meta-analytic level, showing DORs of 60–75 for Raman and MS modalities and ~45 for OCT. Such performance positions these methods within the “excellent” diagnostic category defined for intraoperative molecular diagnostics.

Diagnostic performance differences across modalities are largely explained by inherent trade-offs between spatial resolution, penetration depth, and chemical specificity. Raman-based techniques provide high micrometer-scale resolution and strong biochemical specificity but are limited to superficial tissue sampling. Mass spectrometry offers exceptional molecular specificity and broader tissue sampling, albeit at lower spatial resolution. In contrast, optical coherence tomography enables rapid, deeper structural imaging but lacks direct chemical contrast, which may limit sensitivity at infiltrative margins. These physical constraints account for the observed modality-specific performance and underscore their complementary roles in intraoperative glioma assessment.

### 4.2. Tumor vs. Normal Tissue

The primary endpoint—differentiation of tumor from normal brain tissue—showed pooled sensitivity of 0.90, specificity of 0.88, and a DOR of72.4, confirming exceptional accuracy across all technologies. Raman and MS methods, which measure biochemical composition directly, outperformed OCT, which is limited to morphological contrast. These results reinforce that chemical specificity rather than structural imaging alone underlies the diagnostic power of optical and molecular spectroscopy [14,20]. Notably, the low heterogeneity (I^2^ = 26%) across these studies indicates stable diagnostic reliability irrespective of specific instrumentation.

### 4.3. Infiltrated vs. Non-Infiltrated Margins

Detection of infiltrated glioma margins remains one of neurosurgical oncology’s greatest challenges. Even experienced surgeons and pathologists struggle to delineate these regions intraoperatively, given their near-normal appearance and variable cellularity [5]. Here, pooled accuracy for infiltrated vs. non-infiltrated margins (DOR ≈ 42, logDOR ≈ 3.7) demonstrates that spectroscopic and mass-spectrometric approaches can capture subtle biochemical cues indicative of infiltration. For instance, Raman studies have linked elevated lipid-to-protein ratios and nucleic acid vibrational shifts to infiltrative margins [13,28], while MS-based techniques detect gradient changes in phosphatidylcholine and sphingomyelin species [18,19]. Although this diagnostic task remains less accurate than binary tumor vs. normal differentiation, the achieved performance is clinically meaningful and far surpasses visual inspection or MRI guidance alone [9].

### 4.4. Molecular Stratification: IDH-Mutant vs. Wild-Type Gliomas

A key novel insight from this meta-analysis is that optical and spectrometric modalities can enable intraoperative molecular classification. Across seven studies assessing IDH status, pooled sensitivity and specificity were 0.87 and 0.85, with a DOR of 52.3 (logDOR = 3.96). This aligns with recent advances in DESI-MS and Raman techniques showing direct detection of the oncometabolite 2-hydroxyglutarate (2HG), a hallmark of IDH-mutant gliomas [37]. Maitra et al. [21] demonstrated that machine-learning classifiers trained on MS spectra could predict IDH genotype in <2 min per sample, achieving 94% accuracy. These findings suggest that intraoperative molecular phenotyping—long a post-operative process—may soon be available in real time, guiding resection strategy and even intraoperative enrollment in molecularly stratified clinical trials [12].

### 4.5. Heterogeneity and Bias Assessment

Moderate heterogeneity (I^2^ ≈ 38%) across all analyses primarily reflects differences in acquisition settings, data processing, and patient cohorts. Nevertheless, the direction of effect was consistent across modalities, and no single study significantly skewed pooled estimates. Importantly, funnel plot analysis of logDOR values revealed symmetrical distribution around the pooled mean (≈4.2), while both Egger’s regression and Deeks’ asymmetry test were non-significant (*p* > 0.10), indicating an absence of publication bias. Quality appraisal via QUADAS-2 confirmed low to moderate overall risk, with most studies employing blinded reference standards and consecutive sampling. These findings strengthen the validity and generalizability of the conclusions.

### 4.6. Clinical Implications

From a neurosurgical perspective, these results underscore the clinical readiness of spectroscopic and molecular modalities. When integrated into the operative workflow, technologies such as SRH and iKnife can reduce dependence on frozen section analysis, improve the extent of resection, and minimize iatrogenic injury by providing objective, continuous feedback at the resection margin [22,44,45]. Moreover, the capacity to simultaneously provide molecular information (e.g., IDH, MGMT) opens up avenues for intraoperative adaptation of surgical strategy and early stratification for adjuvant therapies [3,31]. As systems become more compact and AI-assisted analysis matures, these modalities are poised to become part of the routine armamentarium for real-time neuropathologic assessment.

## 5. Limitations and Future Directions

While this meta-analysis comprehensively integrates available evidence, several limitations warrant mention. First, inter-study heterogeneity remains partly attributable to methodological variation, particularly in algorithm training and spectral preprocessing pipelines. Second, smaller ex vivo studies may overestimate diagnostic performance due to controlled sampling conditions and the absence of intraoperative confounders such as motion or hemostasis. Third, long-term outcome data linking intraoperative spectroscopic margin assessment to survival or recurrence remain limited. Future multicenter trials should standardize data acquisition protocols, incorporate blinded cross-validation, and include prospective endpoints such as the extent of resection, functional outcomes, and progression-free survival.

Although OCT is not a spectroscopic technique, it was included due to its established intraoperative use for real-time margin visualization and its complementary structural information relative to chemically specific spectroscopic methods.

Finally, integration with emerging technologies such as AI-based multimodal data fusion—combining Raman, MS, and OCT signals—could enable real-time multimodal decision support in the surgical field [30,31]. Such advances may transform intraoperative diagnostics from an interpretive adjunct to a quantitative guidance platform, aligning surgical precision with molecular neuropathology.

## 6. Conclusions

This comprehensive meta-analysis demonstrates that Raman spectroscopy, mass spectrometry, and optical coherence techniques achieve excellent real-time diagnostic performance for glioma detection, infiltration assessment, and molecular subtype differentiation. The pooled diagnostic odds ratio of 65.7 (logDOR ≈ 4.18) reflects robust and reproducible accuracy across diverse patient cohorts and instrumentation. These findings establish a solid evidence base for the integration of spectroscopic and molecular modalities into modern neurosurgical practice, representing a paradigm shift toward real-time, molecularly informed surgery.

## Figures and Tables

**Table 2:** Comparison of subcategory diagnostic performance.

Diagnostic Endpoint	Sensitivity	Specificity	DOR	logDOR	I^2^ (%)	Interpretation
Tumor vs. normal tissue	0.91	0.88	72.4	4.28	26	Excellent, stable accuracy
Infiltrated vs. non-infiltrated	0.86	0.82	41.8	3.73	41	Moderate accuracy, higher heterogeneity
IDH-mutant vs. wild-type	0.87	0.85	52.3	3.96	29	High molecular classification accuracy

## Data Availability

The raw data supporting the conclusions of this article will be made available by the authors on request.
